# Supplementary material for: Adaptive immune responses to vaccination reflect social status at first exposure in female rhesus macaques
Source: bioRxiv. 2026 Mar 5:2026.03.03.709117. Preprint. [Version 1] doi: 10.64898/2026.03.03.709117 (PMC12991115; doi:10.64898/2026.03.03.709117)
Supplement: 2 [file NIHPP2026.03.03.709117v1-supplement-2.pdf]

bioRxiv preprint doi: https://doi.org/10.64898/2026.03.03.709117; this version posted March 5, 2026. The copyright holder for this preprint (which was not certified by peer review) is the author/funder, who has granted bioRxiv a license to display the preprint in perpetuity. It is made available under aCC-BY 4.0 International license.

Supplementary Materials for

Adaptive immune responses to vaccination reflect social status at first exposure in female rhesus macaques

Joao Barroso-Batista *et al.*

.

Corresponding authors: Luis B. Barreiro, lbarreiro@uchicago.edu; Jenny Tung, jtung@eva.mpg.de

This PDF file includes:

Figs. S1 to S5
Legends for Data S1 to S8

Other Supplementary Materials for this manuscript include the following:
Data S1 to S8

bioRxiv preprint doi: https://doi.org/10.64898/2026.03.03.709117; this version posted March 5, 2026. The copyright holder for this preprint (which was not certified by peer review) is the author/funder, who has granted bioRxiv a license to display the preprint in perpetuity. It is made available under aCC-BY 4.0 International license.

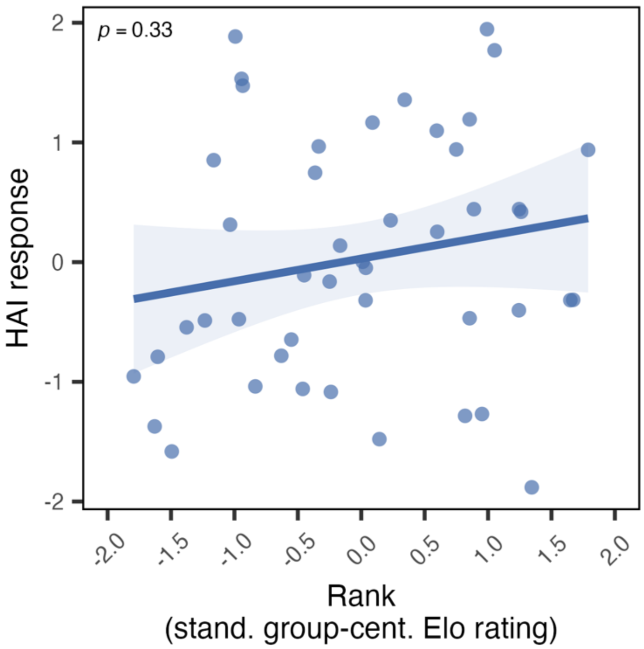

Fig. S1. Effect of dominance rank, measured by Elo rating, on the HAI antibody response (linear mixed model $p = 0.33$). Partial residuals plot for HAI titers (y-axis) as a function of dominance rank, adjusted for the contribution of age, body mass, group membership, and kinship.

bioRxiv preprint doi: https://doi.org/10.64898/2026.03.03.709117; this version posted March 5, 2026. The copyright holder for this preprint (which was not certified by peer review) is the author/funder, who has granted bioRxiv a license to display the preprint in perpetuity. It is made available under aCC-BY 4.0 International license.

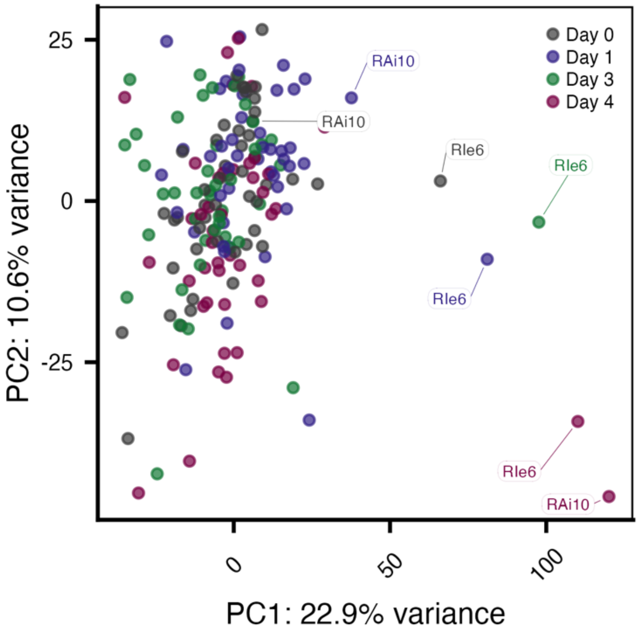

Fig. S2. Principal component analysis of gene expression data across all 4 timepoints. We excluded two individuals who were conspicuous outliers (after batch correction) from subsequent analysis. Samples from the two individuals (Ai10 and Ie6) are labeled above.

bioRxiv preprint doi: https://doi.org/10.64898/2026.03.03.709117; this version posted March 5, 2026. The copyright holder for this preprint (which was not certified by peer review) is the author/funder, who has granted bioRxiv a license to display the preprint in perpetuity. It is made available under aCC-BY 4.0 International license.

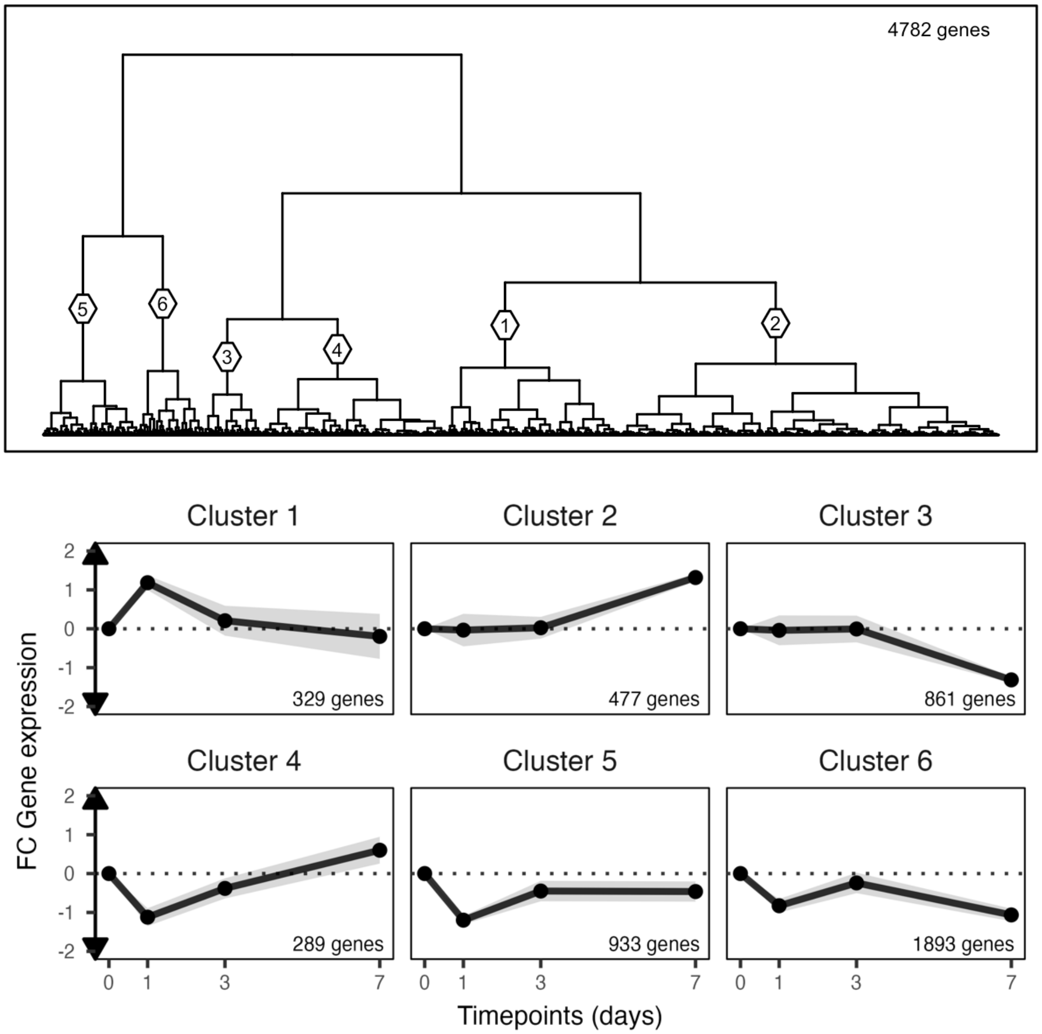

Fig. S3. Hierarchical clustering of genes strongly responsive to the vaccine (FDR 1%). Unsupervised clustering of differentially expressed genes upon vaccination into 6 gene clusters that share similar transcriptional temporal dynamics. We focus on Clusters 1, 2, and 3 in the main text because we identified no enrichments for immune processes in Clusters 4, 5, or 6.

bioRxiv preprint doi: https://doi.org/10.64898/2026.03.03.709117; this version posted March 5, 2026. The copyright holder for this preprint (which was not certified by peer review) is the author/funder, who has granted bioRxiv a license to display the preprint in perpetuity. It is made available under aCC-BY 4.0 International license.

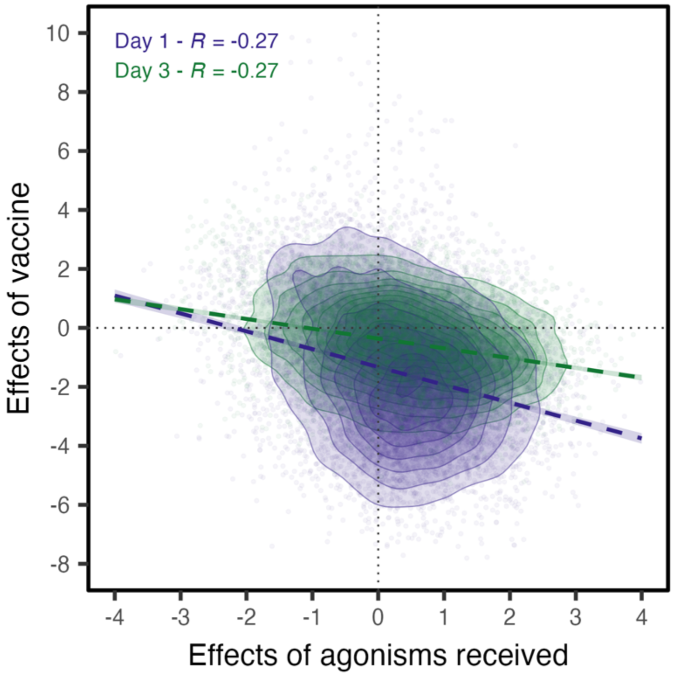

Fig. S4. Correlation between the effects of influenza vaccine and agonisms received on gene expression. Correlation of gene-level effect sizes (standardized betas) between the vaccine response and agonisms received at days 1 and 3 following vaccination (day 7 shows the strongest correlation; see Figure 3B).

bioRxiv preprint doi: https://doi.org/10.64898/2026.03.03.709117; this version posted March 5, 2026. The copyright holder for this preprint (which was not certified by peer review) is the author/funder, who has granted bioRxiv a license to display the preprint in perpetuity. It is made available under aCC-BY 4.0 International license.

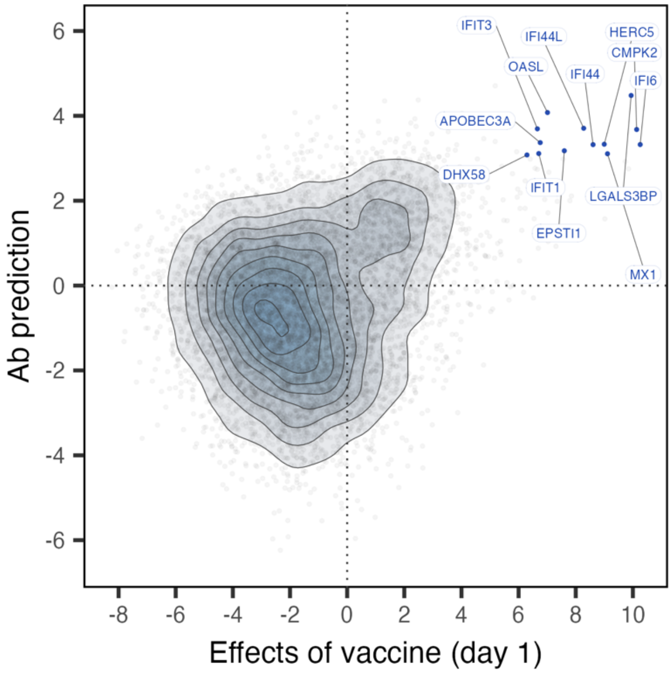

Fig. S5. Correlation between effects of influenza vaccine and antibody prediction. Correlation of gene-level effect sizes (standardized betas) between the vaccine response on day 1 and the relationship between gene expression fold change and the fold change antibody response from baseline to day 28. ISG genes that positively predict the antibody response and that are strongly upregulated 1 day after vaccination tend to fall in the extreme upper right quadrant and are labeled with the gene name.

bioRxiv preprint doi: https://doi.org/10.64898/2026.03.03.709117; this version posted March 5, 2026. The copyright holder for this preprint (which was not certified by peer review) is the author/funder, who has granted bioRxiv a license to display the preprint in perpetuity. It is made available under aCC-BY 4.0 International license.

Data S1 (Microsoft Excel Format). Study subject information and behavioral data.

Data S2 (Microsoft Excel Format). Titers of vaccine-specific IgG antibodies (IgG, log_{10}) and haemagglutination-protective serum antibodies (HAI, log_2) for individuals in phases 1 (IgG, HAI) and 2 (IgG).

Data S3 (Microsoft Excel Format). Full parameter estimates, p-values, and FDR-corrected p-values by gene for flu vaccine effects per timepoint.

Data S4 (Microsoft Excel Format). Assignments of vaccine responsive genes (FDR 1%) to clusters that share similar temporal gene expression dynamics post-vaccination, as determined by hierarchical clustering.

Data S5 (Microsoft Excel Format). Over-representation analysis results (FDR 1%) - clusters of genes that respond to influenza vaccine (differentially expressed genes, FDR 1%).

Data S6 (Microsoft Excel Format). Full parameter estimates, p-values, and FDR-corrected p-values by gene for agonism received effects, within vaccine timepoint.

Data S7 (Microsoft Excel Format). Gene set enrichment analysis results (FDR 1%) for the effects of agonisms received at different timepoints post-vaccination.

Data S8 (Microsoft Excel Format). Gene set enrichment analysis results (FDR 1%) for genes where the fold-change response to vaccination predicts the foldchange antibody response at day 28 post-vaccination.
